# Efficacy and safety of PEGylated exenatide injection (PB-119) in treatment-naive type 2 diabetes mellitus patients: a Phase II randomised, double-blind, parallel, placebo-controlled study

**DOI:** 10.1007/s00125-021-05392-9

**Published:** 2021-03-09

**Authors:** Linong Ji, Ying Du, Min Xu, Xiangjun Zhou, Zhaohui Mo, Jianhua Ma, Jiarui Li, Yufeng Li, Jingna Lin, Yanjun Wang, Jing Yang, Weihong Song, Hui Jin, Shuguang Pang, Hui Liu, Ping Li, Jie Liu, Minxiu Yao, Wenhui Li, Xiaohong Jiang, Feixia Shen, Houfa Geng, Haifeng Zhou, Jianmin Ran, Minxiang Lei, Yinghong Du, Shandong Ye, Qingbo Guan, Wenshan Lv, Huiwen Tan, Tao Chen, Jinkui Yang, Guijun Qin, Shiyun Li, Lei Chen

**Affiliations:** 1 Department of Endocrinology, Peking University People’s Hospital, Beijing, China; 2 PegBio Co., Ltd, Suzhou, China; 3 Department of Endocrinology, The Third Xiangya Hospital of Central South University, Changsha, China; 4 Department of Endocrinology, Nanjing First Hospital, Nanjing, China; 5 The Third Endocrinology Department, Cangzhou Central Hospital, Cangzhou, China; 6 Department of Endocrinology, Beijing Pinggu Hospital, Beijing, China; 7 Department of Endocrinology, Tianjin People’s Hospital, Tianjin, China; 8 Department of Endocrinology, The Second Hospital of Jilin University, Changchun, China; 9 Department of Endocrinology, First Hospital of Shanxi Medical University, Taiyuan, China; 10 Department of Endocrinology and Diabetes, Chenzhou No 1 People’s Hospital, Chenzhou, China; 11 Department of Endocrinology, Zhongda Hospital Southeast University, Nanjing, China; 12 Department of Endocrinology, Jinan Central Hospital, Jinan, China; 13 Department of Endocrinology, Luoyang Central Hospital, Luoyang, China; 14 Department of Endocrinology, Yuncheng Central Hospital, Yuncheng, China; 15 Department of Endocrinology, The First Affiliated Hospital of Henan University of Science and Technology, Henan, China; 16 Department of Endocrinology, Qingdao Central Hospital, Qingdao, China; 17 Department of Endocrinology, Beijing Union Medical College Hospital, Beijing, China; 18 Department of Endocrinology, The First People’s Hospital of Changzhou, Changzhou, China; 19 Department of Endocrinology, The First Affiliated Hospital of Wenzhou Medical University, Wenzhou, China; 20 Department of Endocrinology, Xuzhou Central Hospital, Xuzhou, China; 21 Department of Endocrinology, The First People’s Hospital, Changde, China; 22 Department of Endocrinology, Guangzhou Red Cross Hospital, Guangzhou, China; 23 Department of Endocrinology, Xiangya Hospital Central South University, Changsha, China; 24 Department of Endocrinology, Guangzhou Panyu Central Hospital, Guangzhou, China; 25 Department of Endocrinology, Anhui Provincial Hospital, Hefei, China; 26 Department of Endocrinology, Shandong Provincial Hospital, Jinan, China; 27 Department of Endocrinology, The Affiliated Hospital of Qingdao University, Qingdao, China; 28 Department of Endocrinology, West China Hospital Sichuan University, Sichuan, China; 29 Department of Endocrinology, Beijing Tongren Hospital, CMU, Beijing, China; 30 Department of Endocrinology, The First Affiliated Hospital of Zhengzhou University, Henan, China; 31 Department of Endocrinology, Affiliated Hospital & Clinical Medical College of Chengdu University, Chengdu, China; 32 Department of Endocrinology, Suzhou Municipal Hospital, Suzhou, China

**Keywords:** Long-acting exenatide, Once-weekly exenatide, PB-119, PEGylation, Type 2 diabetes

## Abstract

**Aims/hypothesis:**

Glucagon-like peptide 1 receptor agonists (GLP-1 RA) such as exenatide are used as monotherapy and add-on therapy for maintaining glycaemic control in patients with type 2 diabetes mellitus. The current study investigated the safety and efficacy of once-weekly PB-119, a PEGylated exenatide injection, in treatment-naive patients with type 2 diabetes.

**Methods:**

In this Phase II, randomised, placebo-controlled, double-blind study, we randomly assigned treatment-naive Chinese patients with type 2 diabetes in a 1:1:1:1 ratio to receive subcutaneous placebo or one of three subcutaneous doses of PB-119 (75, 150, and 200 μg) for 12 weeks. The primary endpoint was the change in HbA_1c_ from baseline to week 12, and other endpoints were fasting plasma glucose, 2 h postprandial glucose (PPG), and proportion of patients with HbA_1c_ < 53 mmol/mol (<7.0%) and ≤48 mmol/mol (≤6.5%) at 2, 4, 8 and 12 weeks of treatment. Safety was assessed in all patients who received at least one dose of study drug.

**Results:**

We randomly assigned 251 patients to one of the four treatment groups (*n* = 62 in placebo and 63 each in PB-119 75 μg, 150 μg and 200 μg groups). At the end of 12 weeks, mean differences in HbA_1c_ in the treatment groups were −7.76 mmol/mol (95% CI −9.23, −4.63, *p* < 0.001) (−0.72%, 95% CI −1.01, −0.43), −12.89 mmol/mol (95% CI −16.05, −9.72, *p* < 0.001) (−1.18%, 95% CI −1.47, −0.89) and −11.14 mmol/mol (95% CI −14.19, −7.97, *p* <0 .001) (−1.02%, 95% CI −1.30, −0.73) in the 75 μg, 150 μg and 200 μg PB-119 groups, respectively, compared with that in the placebo group after adjusting for baseline HbA_1c_. Similar results were also observed for other efficacy endpoints across different time points. There was no incidence of treatment-emergent serious adverse event, severe hypoglycaemia or death.

**Conclusions/interpretation:**

All tested PB-119 doses had superior efficacy compared with placebo and were safe and well tolerated over 12 weeks in treatment-naive Chinese patients with type 2 diabetes.

**Trial registration:**

ClinicalTrials.gov NCT03520972

**Funding:**

The study was funded by National Major Scientific and Technological Special Project for Significant New Drugs Development and PegBio.

**Graphical abstract:**

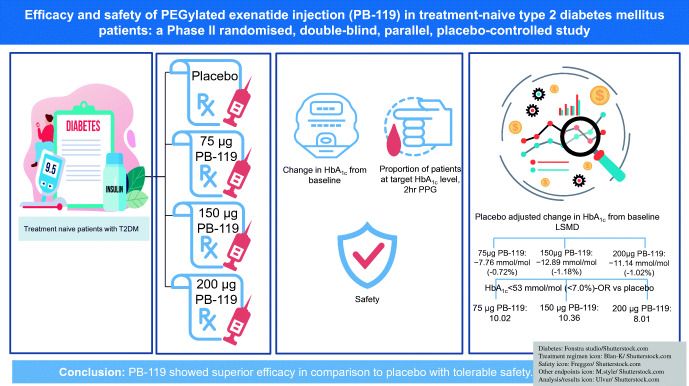

**Supplementary Information:**

The online version of this article (10.1007/s00125-021-05392-9) contains peer-reviewed but unedited supplementary material.

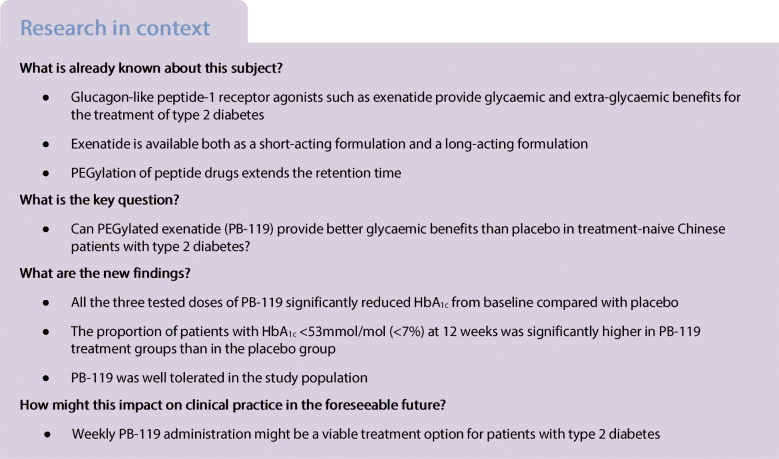



## Introduction

Type 2 diabetes mellitus affects approximately 9.3% of the world population, and is projected to increase to 10.9% by 2045 [1]. Despite the availability of multiple antidiabetic drugs, disease progression and deterioration of glycaemic control are difficult to prevent. Hence, new therapeutic drugs are being pursued [2, 3]. The incretin system is an important target for the therapeutic management of type 2 diabetes [4]. Incretins are intestinal hormones that regulate insulin production in response to oral intake of nutrients, called the ‘incretin effect’, which is lacked in patients with type 2 diabetes [5].

Glucagon-like peptide-1 receptor agonists (GLP-1 RAs) are a novel class of injectable incretin mimetics that provide glycaemic and extra-glycaemic benefits for the treatment of patients with type 2 diabetes [6]. The glycaemic effects of GLP-1 RAs are mainly mediated by the induction of glucose-dependent insulin secretion, inhibition of glucagon secretion, reduction of gastric motility and promotion of satiety [7–11]. Exenatide is a first-in-class GLP-1 RA, available both as a short-acting formulation (twice daily) and as a long-acting (once weekly) formulation [12, 13]. It has been used both as monotherapy with lifestyle modifications and as an add-on therapy with oral antidiabetic drugs (OADs) or insulin in addition to dipeptidyl peptidase-4 inhibitors [14, 15]. The terminal t½ of the original subcutaneous formulation of exenatide was 2.4 h, requiring twice daily injections [16]. In earlier clinical trials with the twice daily formulation, an HbA_1c_ reduction of 1.0% to 1.5% was observed in patients who had not been well controlled with OADs, with a baseline HbA_1c_ of 7.9% (63 mmol/mol) to 9.0% (75 mmol/mol) [17, 18].

The short t½ of exenatide had impeded the routine use of exenatide; this has prompted the use of microsphere technology to extend the t½, creating a feasible once weekly formulation [12]. Exenatide once weekly regimen dispersed by the microsphere technology has been evaluated in previous trials, both as a monotherapy and in combination with OADs and insulin [13, 19–21]. One of its potential limitations is the multiphasic concentration-time profile, which limits the prediction of accurate pharmacokinetic (PK) variables. Meanwhile, the need for a dispersing diluent may affect patients’ compliance [16].

Covalent attachment of polyethylene glycol (PEG) to peptide drugs such as exenatide increases the relative molecular mass and reduces the renal clearance rate, prolonging retention in the circulation [22]. It also reduces the immunogenicity, thereby preventing adverse immunological reactions. The study drug of this study, PEGylated exenatide injection (PB-119), has been previously evaluated for safety, tolerability, and PK effects in a Phase 1 study with 70 healthy volunteers, and the study recommended a once-weekly PB-119 injection of 2 to 200 μg, showing this dose to be safe and well tolerated [23]. In this study, we assessed the efficacy, tolerability and safety of different doses of PB-119 as a monotherapy, compared with placebo, in treatment-naive Chinese patients with type 2 diabetes.

## Methods

### Study design

This Phase II, randomised, multiple doses, double-blind, parallel, placebo-controlled, four-arm study was conducted in 31 clinical centres in China (ClinicalTrials.gov registration no: NCT03520972). The protocol was approved by the institutional review board of the participating study centres. The study was conducted in accordance with the Declaration of Helsinki and other local regulatory guidelines governing the conduct of clinical studies in China. All patients provided written informed consent to participate before study initiation.

### Study population

Eligible patients were men or women with type 2 diabetes (according to 1999 WHO type 2 diabetes diagnostic criteria) who were aged 18 to 70 years, with a BMI of 18.5 to 35 kg/m^2^, and were on a diet and exercise regimen. All patients were previously untreated for 3 months with any antidiabetic drug except short-term insulin treatment (≤7 days). At the time of randomisation, the patients were required to have HbA_1c_ between 58.5 mmol/mol (7.5%), and 91.3 mmol/mol (11.0%) and fasting plasma glucose (FPG) between 4.4 and 13.3 mmol/l.

Key exclusion criteria were pregnancy, clinical diagnosis of type 1 diabetes, acute complications of diabetes, previous incidence of severe hypoglycaemia within 6 months before the study, a severe cardiovascular event within 6 months before screening, any type of malignancy, uncontrolled high BP (systolic BP >160 mmHg or diastolic BP >100 mmHg), haemoglobin concentration of <12 mg/l for men and <10 mg/l for women, severe gastrointestinal diseases, history or ongoing symptoms or signs of severe allergy or hypersensitivity, triacylglycerol concentration >5.65 mmol/l or lipid-lowering drugs used within 3 months before screening, and renal dysfunction (GFR <45 ml min^−1^ [1.73 m]^−2^ according to the Modification of Diet in Renal Disease formula).

### Randomisation and masking

Eligible patients were enrolled into a single-blind run-in period of 2 weeks during which placebo was administered. The patients were then assessed for eligibility again at the end of run-in period. Patients who met the eligibility criteria were randomised by the randomisation plan devised by the SAS 9.4 statistical tool in a 1:1:1:1 to one of the three different doses of PB-119 (75, 150 and 200 μg subcutaneous injection, once weekly) or placebo group. The random grouping information was maintained in the central randomisation system, and each drug was given a specific number. The dosages of PB-119 or placebo were delivered by a weekly, subcutaneous abdominal injection at a dose of 0.5 mg/ml during any time of the day. The investigators, site personnel, patients, and sponsors were masked to treatment assignment, and the anonymised data were stored in a secured directory that was accessible only after the study was unblinded. The participants were distributed across 31 research centres.

### Procedures and data collection

Demographic data were collected during the screening period. Different laboratory variables including HbA_1c_ and FPG were assessed prior to randomisation. Patients were treated for 12 weeks and HbA_1c_, FPG, and 2 h postprandial glucose (PPG) were measured at 4, 8 and 12 weeks.

### Outcomes and endpoints

The primary efficacy endpoint was change in HbA_1c_ between baseline (day 1) and at the end of 12 weeks. The secondary efficacy endpoints included the proportion of participants with HbA_1c_ <53 mmol/mol (<7.0%) and ≤48 mmol/mol (≤6.5%) at the end of 4, 8 and 12 weeks; change in FPG from baseline at 2, 4, 8 and 12 weeks; and 2 h PPG at 4, 8 and 12 weeks. Changes in blood pressure, body weight and lipid profile were also evaluated.

Safety outcomes were assessed by the incidence of adverse events (AE) as defined by the MedDRA version 22.0. The different AEs included incidence of hypoglycaemic events (blood glucose <3.9 mmol/l), clinical findings in the physical examination, vital signs, 12-lead ECG and clinical laboratory tests.

### Statistical analysis

The sample size was determined based on an assumed change in HbA_1c_ after 12 weeks of treatment from a baseline of −8 mmol/mol (−0.8%), −12 mmol/mol (−1.1%) and −12 mmol/mol (−1.1%) in the three treatment groups and 0 mmol/mol (0%) in the placebo group, and the combined SD was assumed to be 13 mmol/mol (1.2%). The two-sided α level was set at 0.05, the Bonferroni method was used to adjust the multiple comparison, and the ratio of participants in the four groups was 1:1:1:1. On the basis of these variables, the number of participants required for 80% statistical power was determined to be 50 patients in each group. Considering a dropout rate of approximately 20%, 240 participants were planned to be enrolled in this study.

The full analysis set (FAS) included all patients who were randomised and who received at least a single dose of the study drug after the run-in phase, with data from at least one post-baseline data. Efficacy analysis was based on the intention-to-treat principle and included all patients who received at least one dose of the study medication and had at least one post-baseline assessment of the primary endpoint. Safety analysis included all randomised patients who received at least one dose of study medication. We assessed the primary endpoint using an ANCOVA model, with study treatment as a fixed effect and baseline HbA_1c_ as a covariate. Least squares mean (LSM), SE and the corresponding 95% CI for each treatment were calculated by the ANCOVA model. The within-group change in HbA_1c_ was assessed by a paired *t* test. Change in HbA_1c_ at 2, 4 and 8 weeks; change in FPG at 2, 4, 8 and 12 weeks; and change in 2 h PPG at 4, 8 and 12 weeks were also analysed by the ANCOVA model and *t* test. The efficacy indicators including observational indicators were imputed from last available post-baseline follow-up data in the case of missing patients/dropouts. The proportion of patients with HbA_1c_ <53 mmol/mol (<7.0%) and ≤48 mmol/mol (≤6.5%) at 4, 8 and 12 weeks was analysed by Fisher’s test.

Descriptive statistics (number of patients, median [IQR], and mean [SD]) were used to summarise continuous variables. Sensitivity analysis for the main outcome with the original observation values was performed using a mixed-effect model repeated measure (MMRM) model. Baseline HbA_1c_ value, group, visit, interaction between group and visit, and interaction between centre, centre and group were included in the MMRM model. Treatment-emergent AEs were assessed in the safety dataset, which included all patients who received at least one dose of the study drug after the run-in phase. All statistical analyses were performed using SAS version 9.4 (SAS Institute, Cary, NC, USA). All the statistical tests were done at a two-sided α level of 0.05.

## Results

**Fig. 1:**
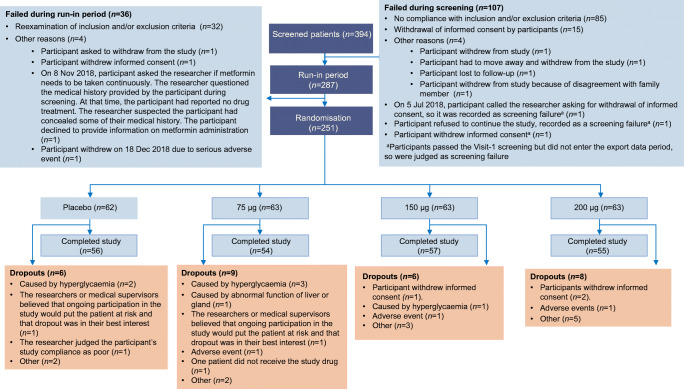
Patient disposition in the study

Between June 2018 and July 2019, we screened 394 participants, of whom 287 eligible patients entered the run-in phase, and 251 patients were randomly assigned to receive 75 μg PB-119 once weekly (*n* = 63), 150 μg PB-119 once weekly (*n* = 63), 200 μg PB-119 once weekly (*n* = 63), and matching placebo group (*n* = 62). Of the 251 randomised patients, except one patient in the 75 μg PB-119 group who did not receive the study drug, all received at least one study dose and entered the FAS. Of the 250 randomised participants, 222 patients completed the study with a minimum of one time point post-baseline follow-up data. A total of 29 patients were withdrawn from the study mainly due to hyperglycaemia (20.7%) and withdrawal of informed consent (10.3%) (Fig. 1). Demographic and disease characteristics at baseline in the FAS are provided in Table 1.

**Table 1:** Demographic characteristics of patients included in the study

Demographic	Categories	Placebo group (*n* = 62)	75 μg PB-119 (*n* = 62)	150 μg PB-119 (*n* = 63)	200 μg PB-119 (*n* = 63)	Total (*N* = 250)	*p* value between the four groups
Age (years)	Number of patients (missing)	62 (0)	62 (0)	63 (0)	63 (0)	250 (0)	
	Mean (SD)	50.7 (10.81)	50.8 (8.93)	51.4 (9.77)	50.5 (10.24)	50.9 (9.91)	0.992
Age stratification, *n* (%)	18–49	30 (48.4)	24 (38.7)	30 (47.6)	26 (41.3)	110 (44)	0.788
	50–70	32 (51.6)	38 (61.3)	33 (52.4)	37 (58.7)	140 (56)	
Sex, *n* (%)	Male	37 (59.7)	48 (77.4)	41 (65.1)	31 (49.2)	157 (62.8)	0.026
	Female	25 (40.3)	14 (22.6)	22 (34.9)	32 (50.8)	93 (37.2)	
Nationality, *n* (%)	Han nationality	62 (100)	61 (98.4)	62 (98.4)	61 (96.8)	246 (98.4)	0.782
	Other	0	1 (1.6)	1 (1.6)	2 (3.2)	4 (1.6)	
Female fertility probability, *n* (%)	Possible pregnancy	13 (52)	2 (14.3)	9 (40.9)	13 (40.6)	37 (39.8)	0.353
	Sterilisation (childbearing age)	0	0	0	0	0	
	Menopause (more than 12 months from the last menstruation)	11 (44)	11 (78.6)	12 (54.5)	19 (59.4)	53 (57)	
	Other	1 (4)	1 (7.1)	1 (4.5)	0	3 (3.2)	
Funduscopy, *n* (%)	Normal	44 (71)	42 (67.7)	41 (65.1)	34 (54)	161 (64.4)	0.346
	Abnormal without clinical significance	11 (17.7)	8 (12.9)	9 (14.3)	10 (15.9)	38 (15.2)	
	Abnormal with clinical significance	7 (11.3)	12 (19.4)	13 (20.6)	19 (30.2)	51 (20.4)	
Course of T2DM (years)	Mean (SD)	2.66 (3.922)	3.20 (4.312)	3.28 (3.949)	3.48 (4.443)	3.16 (4.149)	0.856
Course of T2DM, *n* (%)	≤3 years	49 (79)	41 (66.1)	39 (61.9)	35 (55.6)	164 (65.6)	0.131
	>3 to ≤10 years	10 (16.1)	13 (21)	17 (27)	24 (38.1)	64 (25.6)	
	>10 years	3 (4.8)	8 (12.9)	7 (11.1)	4 (6.3)	22 (8.8)	
Baseline HbA_1c_ (%)	Number of patients (missing)	62 (0)	62 (0)	63 (0)	63 (0)	250 (0)	
	Mean (SD), mmol/mol	72 (5.83)	70 (6.88)	72 (7.81)	71 (6.47)	71 (6.76)	0.705
	Mean (SD), %	8.74 (0.708)	8.57 (0.842)	8.77 (0.951)	8.65 (0.788)	8.68 (0.826)	
	Median, mmol/mol	72	68	72	69	70	
	Median, %	8.75	8.40	8.70	8.50	8.60	
	Minimum, maximum, mmol/mol	58, 91	56, 96	52, 96	55, 98	52, 98	
	Minimum, maximum, %	7.5, 10.5	7.3, 10.9	6.9, 10.9	7.2, 11.1	6.9, 11.1	
	<69 mmol/mol (8.5%)	24 (38.7)	32 (51.6)	27 (42.9)	29 (46)	112 (44.8)	0.315
	≥69 mmol/mol (8.5) to ≤80 mmol/mol (9.5%)	31 (50)	21 (33.9)	20 (31.7)	26 (41.3)	98 (39.2)	
	>80 mmol/mol (9.5%)	7 (11.3)	9 (14.5)	16 (25.4)	8 (12.7)	40 (16)	
Baseline FPG (mmol/l)	Mean (SD)	9.988 (1.7413)	9.681 (2.4607)	10.193 (2.5615)	10.124 (2.4116)	9.998 (2.3123)	0.773
Baseline 2 h PPG	Mean (SD)	16.590 (2.3892)	15.793 (3.6318)	16.829 (4.1007)	16.307 (3.7708)	16.381 (3.5364)	0.564
Other comorbidities		60 (96.8%)	55 (88.7%)	59 (93.7%)	57 (90.5%)	231 (92.4%)	
Hyperlipidaemia		31 (50%)	27 (43.5%)	37 (58.7%)	26 (41.3%)	121 (48.4%)	
Hypertension		25 (40.3%)	19 (30.6%)	30 (47.6%)	22 (34.9%)	96 (38.4%)	
Hepatic steatosis		17 (27.4%)	25 (40.3%)	15 (23.8%)	22 (34.9%)	79 (31.6%)	

T2DM, type 2 diabetes mellitus

### Treatment compliance

The number of patients with a treatment compliance of 80%–100% were: 56 patients (90.3%) in the placebo group, 54 patients (87.1%) in the PB-119 75 μg group, 57 patients (90.5%) in the 150 μg PB-119 group and 56 patients (88.9%) in the 200 μg PB-119 group.

### Efficacy

**Fig. 2:**
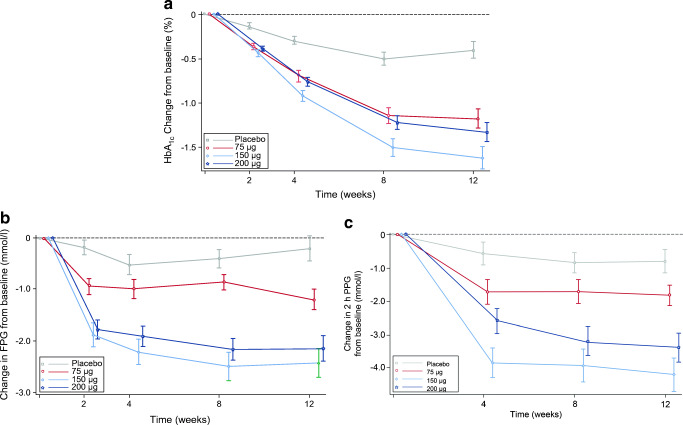
(**a**) Change in HbA_1c_ from baseline at 2, 4, 8 and 12 weeks; (**b**) change in FPG from baseline at 2, 4, 8 and 12 weeks; (**c**) change in 2 h PPG from baseline at 4, 8 and 12 weeks

After 12 weeks of treatment, the LSM change in HbA_1c_ from baseline was −4.26 mmol/mol (95% CI −6.55, −2.07) (−0.39%, 95% CI −0.60, −0.19) in the placebo group, −12.02 mmol/mol (95% CI −14.3, −9.86) (−1.11%, 95% CI −1.32, −0.91) in the 75 μg PB-119 group, −17.15 mmol/mol (95% CI −19.45, −14.97) (−1.57%, 95% CI −1.78, −1.37) in the 150 μg PB-119 group and −15.40 mmol/mol (95% CI −17.59, −13.10) (−1.41%, 95% CI −1.61, −1.20) in the 200 μg PB-119 group. The placebo-adjusted difference in LSM change in HbA_1c_ from baseline to 12 weeks was −7.76 mmol/mol (95% CI −9.23, −4.63) (−0.72%, 95% CI −1.01, −0.43) in the 75 μg PB-119 group, −12.89 mmol/mol (95% CI −16.05, −9.72) (−1.18%, 95% CI −1.47, −0.89) in the 150 μg PB-119 group, and −11.14 mmol/mol (95% CI −14.19, −7.97) (−1.02%, 95% CI −1.30, −0.73) in the 200 μg PB-119 group (Table 2). Although there was a dose-dependent decrease in HbA_1c_ from the 75 μg to 150 μg PB-119 group (LSM difference: −5.13 mmol/mol, 95% CI −8.53, −1.89; −0.46%, 95% CI −0.74, −0.17; *p* = 0.002), there was an increase in HbA_1c_ from the 150 μg to 200 μg PB-119 group (LSM difference: 1.75 mmol/mol, 95% CI −1.24, 4.63; 0.17%, 95% CI, −0.12, 0.45; *p* = 0.261; Table 2). Compared with the placebo group, HbA_1c_ was significantly reduced in all the three dose groups of PB-119 (*p* < 0.001). Similar findings were also observed after 2, 4 and 8 weeks of treatment (Fig. 2a).

**Table 2:** Change in HbA_1c_ from baseline to 12 weeks in treatment groups

	Placebo group (*n* = 62)	75 μg (*n* = 62)	150 μg (*n* = 63)	200 μg (*n* = 63)
Baseline				
Number of patients	62	62	63	63
Mean (SD) (mmol/mol)	72.02 (5.83)	70.16 (6.89)	72.35 (7.85)	71.03 (6.47)
Mean (SD) (%)	8.74 (0.708)	8.57 (0.842)	8.77 (0.951)	8.65 (0.788)
At 12 weeks				
Number of patients	62	62	63	63
Mean (SD) (mmol/mol)	67.54 (7.56)	57.38 (9.65)	54.65 (7.54)	56.50 (8.14)
Mean (SD) (%)	8.33 (0.932)	7.40 (1.245)	7.15 (0.987)	7.32 (1.054)
Changes from baseline to 12 weeks				
Number of patients	62	62	63	63
Mean (SD) (mmol/mol)	−4.48 (8.14)	−12.78 (9.04)	−17.7 (11.08)	−14.53 (8.85)
Mean (SD) (%)	−0.41 (0.745)	−1.18 (0.836)	−1.62 (1.014)	−1.33 (0.810)
*p* value of the four groups	<0.001			
LSM (mmol/mol) (95% CI)	−4.26 (−2.07, 6.55)	−12.02 (−14.3, −9.86)	−17.15 (−19.45, −14.97)	−15.40 (−17.59, −13.10)
LSM % (95% CI)	−0.39 (−0.60, −0.19)	−1.11 (−1.32, −0.91)	−1.57 (−1.78, −1.37)	−1.41 (−1.61, −1.20)
*p* value in the four groups	<0.001	<0.001	<0.001	<0.001
LSMD compared with placebo (mmol/mol) (95% CI)		−7.76 (−9.23, −4.63)	−12.89 (−16.05, −9.72)	−11.14 (−14.19, −7.97)
LSMD compared with placebo (%) (95% CI)		−0.72 (−1.01, −0.43)	−1.18 (−1.47, −0.89)	−1.02 (−1.30, −0.73)
*p* value compared with placebo group		<0.001	<0.001	<0.001
LSMD compared with 75 μg group (mmol/mol) (95% CI)			−5.13 (−8.53, −1.89)	−3.38 (−6.87, 0.00)
LSMD compared with 75 μg group (%) (95% CI)			−0.46 (−0.74, −0.17)	−0.29 (−0.59, 0.00)
*p* value compared with 75 μg group			0.002	0.049
LSMD compared with 150 μg group (mmol/mol) (95% CI)				1.75 (−1.24, 4.63)
LSMD compared with 150 μg group (%) (95% CI)				0.17 (−0.12, 0.45)
*p* value compared with 150 μg group				0.261

LSMD, least squares mean difference

The proportion of patients with HbA_1c_ <53 mmol/mol (<7.0%) at 12 weeks was 8.1% in the placebo group, 46.8% in the 75 μg PB-119 group, 47.6% in the 150 μg PB-119 group and 41.3% in the 200 μg PB-119 group. The OR of achieving HbA_1c_ of <53 mmol/mol (<7.0%) was 10.02 (95% CI 3.54, 28.38), 10.36 (95% CI 3.67, 29.30) and 8.01 (95% CI 2.82, 22.73) in the 75 μg, 150 μg and 200 μg PB-119 groups, respectively, compared with the placebo group (*p* < 0.001). Similar results were also observed for 4 and 8 weeks (Table 3). The proportion of participants with HbA_1c_ ≤ 48 mmol/mol (≤6.5%) at 12 weeks was 1.6% in the placebo group, 27.4% in the 75 μg PB-119 group, 30.2% in the 150 μg PB-119 group and 19.0% in the 200 μg PB-119 group. The OR of achieving HbA_1c_ ≤ 48 mmol/mol (≤6.5%) was 23.04 (95% CI 2.96, 179.59; *p* < 0.001), 26.34 (95% CI 3.40, 204.19; *p* < 0.001) and 14.35 (95% CI 1.80, 114.16; *p* = 0.002) in the 75 μg, 150 μg, and 200 μg PB-119 groups, respectively, compared with the placebo group (*p* < 0.001).

**Table 3:** Proportion of patients with HbA_1c_ <53 mmol/mol (<7%) in the treatment groups

	Placebo group (*n* = 62)	75 μg (*n* = 62)	150 μg (*n* = 63)	200 μg (*n* = 63)
4 weeks				
Number of patients	60	60	58	59
Number of qualified persons	0	10	8	5
Compliance rate (%)	0.0	16.7	13.8	8.5
OR compared with placebo (95% CI)		25.16 (1.44, 439.98)	20.37 (1.15, 361.55)	12.21 (0.66, 225.98)
*p* value^a^ compared with placebo		0.001	0.003	0.027
OR compared with 75 μg group (95% CI)			0.80 (0.29, 2.19)	0.46 (0.15, 1.45)
*p* value^a^ compared with 75 μg group			0.799	0.269
OR compared with 150 μg group (95% CI)				0.58 (0.18, 1.89)
*p* value^a^ compared with 150 μg group				0.394
8 weeks				
Number of patients	57	56	57	57
Number of qualified persons	3	24	23	15
Compliance rate (%)	5.3	42.9	40.4	26.3
OR compared with placebo (95% CI)		13.50 (3.76, 48.43)	12.18 (3.39, 43.68)	6.43 (1.75, 23.67)
*p* value^a^ compared with placebo		<0.001	<0.001	0.004
OR compared with 75 μg group (95% CI)			0.90 (0.43, 1.91)	0.48 (0.22, 1.05)
*p* value^a^ compared with 75 μg group			0.850	0.077
OR compared with 150 μg group (95% CI)				0.53 (0.24, 1.17)
*p* value^a^ compared with 150 μg group				0.164
12 weeks				
Number of patients	62	62	63	63
Number of qualified persons	5	29	30	26
Compliance rate (%)	8.1	46.8	47.6	41.3
OR compared with placebo (95% CI)		10.02 (3.54, 28.38)	10.36 (3.67, 29.30)	8.01 (2.82, 22.73)
*p* value^a^ compared with placebo		<0.001	<0.001	<0.001
OR compared with 75 μg group (95% CI)			1.03 (0.51, 2.09)	0.80 (0.39, 1.62)
*p* value^a^ compared with 75 μg group			>0.999	0.591
OR compared with 150 μg group (95% CI)				0.77 (0.38, 1.56)
*p* value^a^ compared with 150 μg group				0.591

^a^Fisher exact probability was used to compare the two groups

After 12 weeks of treatment, the LSM change in FPG was −0.23 mmol/l (−0.66, 0.20) in the placebo group, −1.20 mmol/l (−1.63, −0.77) in the 75 μg PB-119 group, −2.31 mmol/l (−2.73, −1.89) in the 150 μg PB-119 group and −2.22 mmol/l (−2.65, −1.79) in the 200 μg PB-119 group. The placebo-adjusted difference in LSM change in FPG from baseline to 12 weeks of treatment was −0.97 mmol/l (−1.58, −0.36; *p* = 0.002) in the 75 μg PB-119 group, −2.08 mmol/l (−2.68, −1.47; *p* < 0.001) in the 150 μg PB-119 group, and −1.99 mmol/l (−2.59, −1.39; *p* < 0.001) in the 200 μg PB-119 group. Similar findings were also observed after 2, 4 and 8 weeks of treatment (Fig. 2b). After 12 weeks of treatment, the LSM change in 2 h PPG was −0.76 mmol/l (−1.50, −0.02) in the placebo group, −1.80 mmol/l (−2.53, −1.06) in the 75 μg PB-119 group, −3.96 mmol/l (−4.69, −3.23) in the 150 μg PB-119 group and −3.61 mmol/l (−4.35, −2.87) in the 200 μg PB-119 group. The placebo-adjusted difference in LSM change in 2 h PPG from baseline to 12 weeks of treatment was −1.04 mmol/l (−2.09, 0.02) in the 75 μg PB-119 group, −3.20 mmol/l (−4.25, −2.15) in the 150 μg PB-119 group and −2.85 mmol/l (−3.88, −1.82) in the 200 μg PB-119 group. The reduction in 2 h PPG was significantly higher in the 150 μg and 200 μg PB-119 groups compared with the placebo group (*p* < 0.001) and approaching significance in the 75 μg PB-119 group (*p* = 0.054). Similar findings were also observed after 4 and 8 weeks of treatment (Fig. 2c).

### Changes in BP, body weight and lipid profile

The mean change in systolic and diastolic BP after 12 weeks, were −2.4, −1.5, −2.7 and −2.9 mmHg and −0.7, −0.9, −1.3 and −2.7 mmHg in placebo, 75, 150 and 200 μg PB-119 groups, respectively. The mean changes in body weight and lipids are provided in ESM Table 1.

### Safety

A total of 250 patients received at least one dose of the study drug and constituted the safety dataset. Most of the AEs were mild to moderate. During the treatment period, the incidence of AEs was 69.4% (43 participants) in the placebo group, 77.4% (48 participants) in the 75 μg PB-119 group, 81% (51 participants) in the 150 μg PB-119 group and 82.5% (52 participants) in the 200 μg PB-119 group. The number of AEs in the four groups was 116 in the placebo group, 126 in the 75 μg PB-119 group, 220 in the 150 μg PB-119 group, and 298 in the 200 μg PB-119 group. There were totally 377 events of drug-related AEs reported in 86 patients, with 14 events in six patients in the placebo group, 39 events in 20 patients in the 75 μg PB-119 group, 144 events in 29 patients in the 150 μg PB-119 group and 180 events in 31 patients in the 200 μg PB-119 group (Table 4). There were no deaths or drug-related serious AEs reported in any of the groups. Hypoglycaemia related to the study drug occurred in six (9.7%) patients in the 75 μg PB-119 group, seven (11.1%) patients in the 150 μg PB-119 group and four (6.3%) patients in the 200 μg PB-119 group. Severe hypoglycaemia was not reported in any of the groups. No clinically significant abnormalities in laboratory variables, 12-lead ECG, physical examination or vital signs were observed in any treatment groups.

**Table 4:** Summary of drug-related AEs

	Placebo group	75 μg	150 μg	200 μg
	(*n* = 62)	(*n* = 62)	(*n* = 63)	(*n* = 63)
	*n* (%)	*n* (%)	*n* (%)	*n* (%)
	[events]	[events]	[events]	[events]
Drug-related AEs	6 (9.7) [14]	20 (32.3) [39]	29 (46.0) [144]	31 (49.2) [180]
Serious drug association	0	0	0	0
AEs leading to withdrawal from the study	0	1 (1.6) [2]	1 (1.6) [1]	1 (1.6) [3]
Gastrointestinal AEs	1 (1.6) [1]	5 (8.1) [11]	17 (27.0) [40]	25 (39.7) [110]
Nausea	0	4 (6.5) [4]	10 (15.9) [11]	21 (33.3) [74]
Vomiting	0	1 (1.6) [1]	8 (12.7) [8]	14 (22.2) [24]
Nervous system AEs	1 (1.6) [1]	5 (8.1) [5]	7 (11.1) [64]	8 (12.7) [18]
Laboratorial anomalies	3 (4.8) [11]	6 (9.7) [12]	5 (7.9) [7]	4 (6.3) [5]
Elevated lipase	0	3 (4.8) [4]	2 (3.2) [2]	3 (4.8) [4]
Elevated amylase	1 (1.6) [1]	1 (1.6) [1]	1 (1.6) [1]	0
Elevated alanine amino transferase	1 (1.6) [1]	0	1 (1.6) [1]	0
Elevated aspartate amino transferase	1 (1.6) [1]	0	0	0
Hypoglycaemia	0	6 (9.7) [8]	7 (11.1) [11]	4 (6.3) [7]
Systemic diseases and various administration site reactions	0	1 (1.6) [1]	5 (7.9) [10]	4 (6.3) [8]
Infection and infectious diseases	0	1 (1.6) [1]	1 (1.6) [1]	1 (1.6) [1]
Hepatobiliary AEs	1 (1.6) [1]	0	1 (1.6) [1]	0
Abnormal liver functions tests	0	0	1 (1.6) [1]	0
Hyperbilirubinaemia	1 (1.6) [1]	0	0	0

### Sensitivity analysis

Sensitivity analysis by MMRM revealed a statistically significant difference in placebo-adjusted change in HbA_1c_ levels in all three PB-119 treatment groups (*p* < 0.001). The change in HbA_1c_ from baseline was not significantly different between the 200 μg PB-119, 75 μg PB-119 (*p* = 0.181), as well as 200 μg PB-119 group and 150 μg PB-119 (*p* = 0.052) groups. The effect estimates and the corresponding 95% CIs are provided in ESM Table 2. Similar results in MMRM and ANCOVA model confirms the robustness of the analysis.

### Immunogenicity

At baseline, PB-119 antibody was identified in 3.2% (two patients) and 1.6% (one patient) of participants in the placebo group and 75 μg PB-119 group, respectively. After 12 weeks of treatment or termination of visit, the positive rates of PB-119 antibody were 3.2% (2 patients) in the placebo group, 22.6% (14 patients) in the 75 μg group, 25.4% (16 patients) in the 150 μg group and 34.9% (22 patients) in the 200 μg group.

## Discussion

The main objective of the current study was to assess the efficacy and safety of PB-119 in treatment-naive patients with type 2 diabetes and to identify the relative efficacies of three different doses of PB-119. The results revealed the superior efficacy of PB-119 compared with placebo, and we also observed dose-dependent efficacy up to 150 μg of PB-119, but there was no significant improvement in efficacy at 200 μg of PB-119 compared with 150 μg of PB-119. The results were consistent across different glycaemic endpoints. We found a favourable safety profile after 12 weeks of treatment with no incidence of drug-related serious AEs or severe hypoglycaemia.

In a previous Phase I study assessing the safety and PK/pharmacodynamics of PB-119, the mean peak retention time was found to be between 20 and 40 h, with an elimination t½ of 45–64 h, supporting the once-a-week administration. There was also not much difference in t½ and retention time among the different doses tested in healthy volunteers without any sex-based difference. The clinical laboratory variables, vital signs, ECG and AEs showed that a single dose of 2–200 μg was safe and tolerable. Even after 6 weeks of administration, PB-119 had a long t½ in vivo with a strong correlation between dose and pharmacokinetic variables. After a single subcutaneous dose of 25–400 μg, the maximum serum concentrations achieved ranged from 7 ng/ml to 99 ng/ml with a time to maximum concentration ranging from 19 h to 34 h [23]. In the case of exenatide twice daily, the maximum concentration that was achieved after a dose of 2.5–5 μg was 0.056–0.085 ng/ml with a time to reach maximum serum concentration of 2 h [24]. In the case of PB-119, the steady state concentrations were reached after 2 weeks while with exenatide once weekly and once monthly suspension, steady state concentrations were reported after 6 weeks [9]. The clinical laboratory parameters, vital signs, ECG and AEs showed that a single dose of PB-119 within the range of 2–200 μg was safe and tolerable.

The findings of our study were consistent with previous placebo-controlled studies with exenatide twice daily and exenatide once weekly (microsphere technology). In the current study, LSM difference of change in HbA_1c_ with reference to the placebo arm was significantly better in the PB-119-treated groups, ranging from −7.76 mmol/mol (−0.72%) to −12.89 mmol/mol (−1.18%). In the early clinical trial with exenatide twice daily, the placebo-adjusted change in HbA_1c_ from baseline ranged from −0.98% to −0.58% in patients previously treated with OADs [17]. The results of our study suggest that PB-119 may have retained similar if not superior glycaemic control. Currently, Phase II dose-finding studies for PB-119 in combination with metformin in patients with HbA_1c_ > 58 mmol/mol (>7.5%) after treatment with metformin have been completed (NCT03604419).

The main advantage of exenatide once weekly is the improved patient adherence because of less frequent dosing. In addition, previous studies have established the superior glycaemic control of exenatide once weekly. In a previous study by Drucker et al., after 30 weeks of treatment in treatment-naive patients with type 2 diabetes, exenatide once weekly had significantly greater change in HbA_1c_ from baseline than exenatide twice daily did (−1.9% vs −1.5%; *p* = 0.0023). Glycaemic control with respect to the proportion of patients with HbA_1c_ < 53 mmol/mol (<7.0%) was also significantly higher in the exenatide once weekly group (77% vs 61%; *p* = 0.0039). Further, the activity of exenatide once weekly was also found to be higher than that of exenatide twice daily in patients with baseline HbA_1c_ > 75 mmol/mol (>9.0%) [19]. The superior efficacy of exenatide once weekly both as monotherapy and as an add-on therapy to OADs and basal insulin was confirmed in the DURATION trials [13]. Considering the mechanism of action of PB-119, which is similar to exenatide QW, PB-119 could also reveal superior efficacy in comparison with available OADs.

In the current study, all PB-119 doses led to clinically meaningful improvement in glycaemic control. Unlike exenatide once weekly and exenatide once-monthly dosing regimens [9], dose-dependent improvement in glycaemic control with PB-119 once weekly seems to attain a plateau at a dose of 150 μg. This suggests a dose of 150 μg to be optimum for Phase III studies. PB-119 also did not lead to significant drug-related hypoglycaemic events, so adjustments in drug dose may not be required for administration, and it could be administered at any time of the day, irrespective of whether the patients are in fasting or fed condition. The AEs with PB-119 suggested that drug titrations is not necessary in patients with different comorbidities. Similar to exenatide once weekly, PB-119 could also be made available as single-use disposable cartridges that may improve patient compliance. PB-119 was reported to be absorbed slowly and have a longer retention time (t½ of ⁓64 h) with low predicted immunogenicity. This is facilitated by the PEGylation technology, which converts small peptides into peptides of larger size, leading to slower renal clearance rates. The utility of PEGylation technology in the treatment of type 2 diabetes was previously explored in the PEGylation of basal insulin [25].

The most well-established AEs in patients treated with GLP-1RAs, as per previous studies were gastrointestinal symptoms and injection-site reactions [26, 27]. In previous studies with GLP-1RAs, nausea was the most predominant AE, which improved with the continuation of study drug [28, 29]. In the current study, gastrointestinal AEs were more common in the 200 μg group, which could be due to the dose-dependent effect. In a previous meta-analysis, the odds of incidence of nausea was higher in patients receiving exenatide 10 μg twice daily than in patients receiving exenatide 5 μg twice daily (OR: 2.28) and exenatide once weekly (OR: 2.78) [30]. This substantiates the dose-dependent increase in gastrointestinal AEs in patients treated with higher doses of exenatide. This increase in nausea may also reduce treatment compliance, which consequently may affect the efficacy of exenatide. This could also contribute to the lack of dose-dependent changes in efficacy from 150 μg to 200 μg PB-119 [31]. The incidence of treatment discontinuation due to AEs was 0% in the placebo group and 1.6% each in 75 μg, 150 μg and 200 μg PB-119 groups, which is much lower than the reported treatment discontinuation rates due to AEs in previous studies with exenatide (up to 4%) [31].

Treatment with PB-119 also significantly reduced total cholesterol and triacylglycerols (150 μg and 200 μg groups, *p*< 0.05), body weight and LDL-C (200 μg group, *p* < 0.05). These findings need to be further evaluated in larger Phase III trials. Further, in the current study, 20–30% of the patients were also positive for anti-exenatide antibodies. In a previous study, 45% of the patients treated with exenatide once weekly were found to be positive for low-titre antibodies, which is much higher than reported in the current study. Nevertheless, apart from injection-site reactions, anti-exenatide antibodies have not been reported to affect the safety or efficacy of the drug [26]. The immunogenicity of PB-119 needs to be substantiated in Phase III trials. In the current study, the benefits of PB-119 in patients with different baseline HbA_1c_ levels (subgroups) were also not assessed and should be assessed in Phase III trials. Further, this study was conducted in Chinese patients, and the efficacy and safety results might not be generalisable to other geographic regions. Moreover, the sample size in the current study was determined for the primary endpoint (change in HbA_1c_), so evaluating PB-119 in a larger patient population over a longer treatment duration may provide further insights. Similarly, the safety events reported in the current study were after a short treatment duration of 12 weeks. Hence further studies with long-term follow-up are required to substantiate our results. Nevertheless, PB-119 showed superior glycaemic control compared with placebo, and we identified 150 μg PB-119 once weekly to be the minimum effective dose with an acceptable safety profile. This needs to be further substantiated in larger Phase III studies investigating PB-119 as a monotherapy in drug-naive patients or as an add-on therapy for patients on OAD and insulin treatment.

To conclude, the tested doses of subcutaneous PB-119 once weekly were found to be an effective treatment option in treatment-naive patients with type 2 diabetes, providing glycaemic benefits with good overall safety and tolerance. The ease of use without titration requirement may provide additional advantages in real-world settings.

## Supplementary Information

ESM Tables (PDF 137 kb)

## Data Availability

Study data will be made available by the corresponding author on reasonable request.

## Abbreviations

AE: Adverse events
FAS: Full analysis set
FPG: Fasting plasma glucose
GLP-1 RAs: Glucagon-like peptide-1 receptor agonists
LSM: Least squares mean
MMRM: Mixed-effect model repeated measure
OADs: Oral antidiabetic drugs
PB-119: PEGylated exenatide injection
PEG: Polyethylene glycol
PK: Pharmacokinetic
PPG: Postprandial glucose
