# Correction to: Efficacy and safety of PEGylated exenatide injection (PB-119) in treatment-naive type 2 diabetes mellitus patients: a Phase II randomised, double-blind, parallel, placebo-controlled study

**DOI:** 10.1007/s00125-021-05460-0

**Published:** 2021-05-26

**Authors:** Linong Ji, Ying Du, Min Xu, Xiangjun Zhou, Zhaohui Mo, Jianhua Ma, Jiarui Li, Yufeng Li, Jingna Lin, Yanjun Wang, Jing Yang, Weihong Song, Hui Jin, Shuguang Pang, Hui Liu, Ping Li, Jie Liu, Minxiu Yao, Wenhui Li, Xiaohong Jiang, Feixia Shen, Houfa Geng, Haifeng Zhou, Jianmin Ran, Minxiang Lei, Yinghong Du, Shandong Ye, Qingbo Guan, Wenshan Lv, Huiwen Tan, Tao Chen, Jinkui Yang, Guijun Qin, Shiyun Li, Lei Chen

**Affiliations:** 1 Department of Endocrinology, Peking University People’s Hospital, Beijing, China; 2 PegBio Co., Ltd, Suzhou, China; 3 Department of Endocrinology, The Third Xiangya Hospital of Central South University, Changsha, China; 4 Department of Endocrinology, Nanjing First Hospital, Nanjing, China; 5 The Third Endocrinology Department, Cangzhou Central Hospital, Cangzhou, China; 6 Department of Endocrinology, Beijing Pinggu Hospital, Beijing, China; 7 Department of Endocrinology, Tianjin People’s Hospital, Tianjin, China; 8 Department of Endocrinology, The Second Hospital of Jilin University, Changchun, China; 9 Department of Endocrinology, First Hospital of Shanxi Medical University, Taiyuan, China; 10 Department of Endocrinology and Diabetes, Chenzhou No 1 People’s Hospital, Chenzhou, China; 11 Department of Endocrinology, Zhongda Hospital Southeast University, Nanjing, China; 12 Department of Endocrinology, Jinan Central Hospital, Jinan, China; 13 Department of Endocrinology, Luoyang Central Hospital, Luoyang, China; 14 Department of Endocrinology, Yuncheng Central Hospital, Yuncheng, China; 15 Department of Endocrinology, The First Affiliated Hospital of Henan University of Science and Technology, Henan, China; 16 Department of Endocrinology, Qingdao Central Hospital, Qingdao, China; 17 Department of Endocrinology, Beijing Union Medical College Hospital, Beijing, China; 18 Department of Endocrinology, The First People’s Hospital of Changzhou, Changzhou, China; 19 Department of Endocrinology, The First Affiliated Hospital of Wenzhou Medical University, Wenzhou, China; 20 Department of Endocrinology, Xuzhou Central Hospital, Xuzhou, China; 21 Department of Endocrinology, The First People’s Hospital, Changde, China; 22 Department of Endocrinology, Guangzhou Red Cross Hospital, Guangzhou, China; 23 Department of Endocrinology, Xiangya Hospital Central South University, Changsha, China; 24 Department of Endocrinology, Guangzhou Panyu Central Hospital, Guangzhou, China; 25 Department of Endocrinology, Anhui Provincial Hospital, Hefei, China; 26 Department of Endocrinology, Shandong Provincial Hospital, Jinan, China; 27 Department of Endocrinology, The Affiliated Hospital of Qingdao University, Qingdao, China; 28 Department of Endocrinology, West China Hospital Sichuan University, Sichuan, China; 29 Department of Endocrinology, Beijing Tongren Hospital, CMU, Beijing, China; 30 Department of Endocrinology, The First Affiliated Hospital of Zhengzhou University, Henan, China; 31 Department of Endocrinology, Affiliated Hospital & Clinical Medical College of Chengdu University, Chengdu, China; 32 Department of Endocrinology, Suzhou Municipal Hospital, Suzhou, China

**Correction to: Diabetologia**

10.1007/s00125-021-05392-9

The following sentence in the Discussion was inappropriately changed to a definite statement during editing: ‘Considering the mechanism of action of PB-119, which is similar to exenatide QW, PB-119 also showed superior efficacy compared with available OADs.’

The journal would like to revert back to the authors’ original phrasing as follows:

‘Considering the mechanism of action of PB-119, which is similar to exenatide QW, PB-119 could also reveal superior efficacy in comparison with available OADs.’

The original article has been corrected.

