# Supplementary material for: Efficacy and safety of PEGylated exenatide injection (PB-119) in treatment-naive type 2 diabetes mellitus patients: a Phase II randomised, double-blind, parallel, placebo-controlled study
Source: Diabetologia. 2021 Mar 9;64(5):1066–78. doi: 10.1007/s00125-021-05392-9 (PMC8012337; doi:10.1007/s00125-021-05392-9)
Supplement: Supplementary file 1 — (PDF 137 kb) [file 125_2021_5392_MOESM1_ESM.pdf]

ESM table 1. Changes in body weight, low density lipoproteins, high density lipoproteins, triglycerides and total cholesterol at the end of 12 weeks.

	Placebo Group (n = 62)	75μg (n = 62)	150μg (n = 63)	200μg (n = 63)
**Body weight (kg)**				
LSM (95% CI)	−0.67 (−1.25, −0.09)	−0.89 (−1.47, −0.31)	−0.67 (−1.24, −0.10)	−1.48 (−2.06, −0.91)
P value of four groups		0.154		
LSMD compared with placebo (95% CI)		−0.22 (−1.04, 0.60)	0.00 (−0.82, 0.82)	−0.82 (−1.62, −0.01)
P value compared with placebo group		0.594	>0.999	0.047
LSMD compared with 75-μg group (95% CI)			0.22 (−0.58, 1.03)	−0.59 (−1.42, 0.23)
P value compared with 75-μg group			0.22 (−0.58, 1.03)	−0.59 (−1.42, 0.23)
LSMD compared with 150-μg group (95% CI)				−0.82 (−1.63, −0.00)
P value compared with 150-μg group				0.050
**Total cholesterol (mmol/L)**				
LSM (95% CI)	0.07 (−0.10, 0.24)	−0.14 (−0.31, 0.04)	−0.27 (−0.44, −0.10)	−0.36 (−0.53, −0.19)
P value of four groups		0.004		
LSMD compared with placebo (95% CI)		−0.21 (−0.45, 0.04)	−0.34 (−0.58, −0.09)	−0.43 (−0.67, −0.19)

P value compared with placebo group		0.102	0.007	<0.001
LSMD compared with 75-μg group (95% CI)			−0.13 (−0.37, 0.11)	−0.23 (−0.47, 0.02)
P value compared with 75-μg group			0.275	0.073
LSMD compared with 150-μg group (95% CI)				−0.09 (−0.34, 0.15)
P value compared with 150-μg group				0.450
		**Triglycerides (mmol/L)**		
LSM (95% CI)	0.32 (−0.05, 0.68)	0.08 (−0.28, 0.44)	−0.26 (−0.61, 0.10)	−0.29 (−0.65, 0.08)
P value of four groups			0.061	
LSMD compared with placebo (95% CI)		−0.24 (−0.75, 0.28)	−0.57 (−1.09, −0.06)	−0.60 (−1.11, −0.09)
P value compared with placebo group		0.365	0.028	0.021
LSMD compared with 75-μg group (95% CI)			−0.34 (−0.84, 0.17)	−0.36 (−0.88,0.15)
P value compared with 75-μg group			0.189	0.168

LSMD compared with 150-µg group (95% CI)				−0.03 (−0.54, 0.49)
P value compared with 150-µg group				0.919
**High density lipoproteins (mmol/L)**				
LSM (95% CI)	0.02 (−0.03, 0.06)	0.03 (−0.02, 0.07)	0.02 (−0.02, 0.07)	−0.01 (−0.06, 0.03)
P value of four groups		0.582		
LSMD compared with placebo (95% CI)		0.01 (−0.05, 0.07)	0.01 (−0.06, 0.07)	(−0.09, 0.03)
P value compared with placebo group		0.757	0.840	0.326
LSMD compared with 75-µg group (95% CI)			−0.00 (−0.07, 0.06)	−0.04 (−0.10, 0.02)
P value compared with 75-µg group			0.914	0.211
LSMD compared with 150-µg group (95% CI)				−0.04 (−0.10, 0.03)
P value compared with 150-µg group				0.251
**Low density lipoproteins (mmol/L)**				
LSM (95% CI)	−0.02 (−0.15, 0.10)	−0.19 (−0.31, −0.06)	−0.12 (−0.24, 0.01)	−0.24 (−0.36, −0.11)
P value of four groups		0.081		
LSMD compared with placebo (95% CI)		−0.16 (−0.34, 0.01)	−0.10 (−0.27, 0.08)	−0.22 (−0.39, −0.04)

P value compared with placebo group	0.068	0.290	0.015
LSMD compared with 75-µg group (95% CI)		0.07 (−0.11, 0.24)	−0.05 (−0.23, 0.13)
P value compared with 75-µg group		0.435	0.572
LSMD compared with 150-µg group (95% CI)			−0.12 (−0.30, 0.06)
P value compared with 150-µg group			0.178

ESM Table 2. Sensitivity analysis for change in HbA_{1c} from baseline to 12 weeks in repeated measurement mixed linear model

	Placebo Group (n = 62)	75µg (n = 62)	150µg (n = 63)	200µg (n = 63)
At 12 weeks				
LSM mmol/mol (95% CI)	−4.26 (−6.55, −2.07)	−13.10 (−15.37, −10.83)	−18.78 (−21.07, −16.37)	−15.50 (−17.79, −13.09)
LSM % (95% CI)	−0.39 (−0.60, −0.18)	−1.21 (−1.42, −1.00)	−1.72 (−1.93, −1.50)	−1.42 (−1.63, −1.20)
LSMD mmol/mol compared with placebo (95% CI)		−8.84 (−12.07, −5.60)	−14.52 (−17.79, −11.24)	−11.24 (−14.51, −7.97)
LSMD % compared with placebo (95% CI)		−0.82 (−1.12, −0.52)	−1.33 (−1.63, −1.03)	−1.03 (−1.33, −0.73)
P value compared with placebo group		<0.001	<0.001	<0.001
LSMD mmol/mol compared with 75-µg group (95% CI)			−5.68 (−9.02, −2.23)	−2.14 (−5.19, −1.01)
LSMD % compared with 75-µg group (95% CI)			−0.51 (−0.81, −0.20)	−0.21 (−0.51, −0.10)
P value compared with 75-µg group			0.001	0.181
LSMD mmol/mol compared with 150-µg group (95% CI)				3.28 (0, 6.56)

LSMD % compared with 150-µg group (95% CI)	0.30 (−0.00, 0.60)
P value compared with 150-µg group	0.052

Abbreviations: CI, confidence interval; LSM, lease squared mean; LSMD, least squares mean difference.
